# Differential regulation of actin-activated nucleotidyl cyclase virulence factors by filamentous and globular actin

**DOI:** 10.1371/journal.pone.0206133

**Published:** 2018-11-12

**Authors:** Dorothée Raoux-Barbot, Alexander Belyy, Lina Worpenberg, Sabrina Montluc, Celia Deville, Véronique Henriot, Christophe Velours, Daniel Ladant, Louis Renault, Undine Mechold

**Affiliations:** 1 Unité de Biochimie des Interactions macromoléculaires, Département de Biologie Structurale et Chimie, CNRS UMR 3528, Institut Pasteur, Paris, France; 2 Institute for Integrative Biology of the Cell (I2BC), CEA, CNRS, Univ. Paris-Sud, Université Paris-Saclay, France; 3 Structural Chemistry and Biology team, Institut de Chimie des Substances Naturelles, CNRS, Université Paris-Saclay, Gif-sur-Yvette, France; Universidad de Costa Rica, COSTA RICA

## Abstract

Several bacterial pathogens produce nucleotidyl cyclase toxins to manipulate eukaryotic host cells. Inside host cells they are activated by endogenous cofactors to produce high levels of cyclic nucleotides (cNMPs). The ExoY toxin from *Pseudomonas aeruginosa* (PaExoY) and the ExoY-like module (VnExoY) found in the MARTX (Multifunctional-Autoprocessing Repeats-in-ToXin) toxin of *Vibrio nigripulchritudo* share modest sequence similarity (~38%) but were both recently shown to be activated by actin after their delivery to the eukaryotic host cell. Here, we further characterized the ExoY-like cyclase of *V*. *nigripulchritudo*. We show that, in contrast to PaExoY that requires polymerized actin (F-actin) for maximum activation, VnExoY is selectively activated by monomeric actin (G-actin). These two enzymes also display different nucleotide substrate and divalent cation specificities. *In vitro* in presence of the cation Mg^2+^, the F-actin activated PaExoY exhibits a promiscuous nucleotidyl cyclase activity with the substrate preference GTP>ATP≥UTP>CTP, while the G-actin activated VnExoY shows a strong preference for ATP as substrate, as it is the case for the well-known calmodulin-activated adenylate cyclase toxins from *Bordetella pertussis* or *Bacillus anthracis*. These results suggest that the actin-activated nucleotidyl cyclase virulence factors despite sharing a common activator may actually display a greater variability of biological effects in infected cells than initially anticipated.

## Introduction

Several bacterial pathogens produce nucleotidyl cyclase toxins that are delivered to specific target cells of their host where they are activated to synthetize massive amounts of cyclic nucleotides (cNMPs) that have key regulatory roles in many cellular processes. Accumulation of cNMPs subsequently triggers profound alterations of the physiology of the host target cells. These enzymes are essentially inactive within their native bacterial host in order to prevent detrimental effects of massive cNMP synthesis and become catalytically active only upon reaching the eukaryotic environment of the host target cells. These toxins are typically activated by interacting with a specific eukaryotic cofactor, usually an abundant protein of their hosts. The adenylate cyclase toxins from *Bordetella pertussis* (CyaA) and edema factor from *Bacillus anthracis* (EF) are strongly activated upon interaction with calmodulin [1–5].

We recently discovered that the nucleotidyl cyclase toxin ExoY from *Pseudomonas aeruginosa* (PaExoY) is activated by actin [6] and showed that this is also the case for a rather distant homolog (~38% sequence similarity) of ExoY, VnExoY-like from *Vibrio nigripulchritudo* (named VnExoY from here on), an emerging marine pathogen infecting farmed shrimps in New Caledonia and other regions in the Indo-Pacific [7]. VnExoY is encoded by the virulence-associated plasmid pASFn1 of *V*. *nigripulchritudo* [7, 8]. As many other ExoY homologs, VnExoY is one of multiple effector domains present in MARTX (Multifunctional-Autoprocessing Repeats-in-ToXin) toxins. A similar ExoY homolog from a MARTX toxin of *Vibrio vulnificus* biotype 3 has been shown to be an adenylate cyclase essential for virulence [8, 9]. We suggested the term actin-activated nucleotidyl cyclases (AA-NC) to unite all these ExoY-like enzymes [6].

We previously showed that polymerized actin (F-actin) is required for maximal activation of PaExoY [6]: i) PaExoY activation by actin was strongly antagonized by different G-actin binding proteins that prevent actin spontaneous nucleation or polymerization, or by the polymerization-inhibiting drug latrunculin A. ii) maximal activation of PaExoY by actin-ATP or actin-ADP was correlated with F-actin formation in each nucleotide state. In addition, ExoY-GFP colocalized with F-actin rich structures within cells.

Here, we further characterized VnExoY from *V*. *nigripulchritudo* and showed that in contrast to *P*. *aeruginosa* ExoY, this enzyme is preferentially activated by G-actin. These two enzymes also display different nucleotide substrate and divalent cation specificities. Hence this study indicates that the actin-activated nucleotidyl cyclase virulence factors despite sharing a common mode of action may trigger a much wider range of biological effects on infected cells than initially anticipated.

## Results and discussion

### Differential effects of actin polymerization on the activation of PaExoY and VnExoY

**Fig 1:**
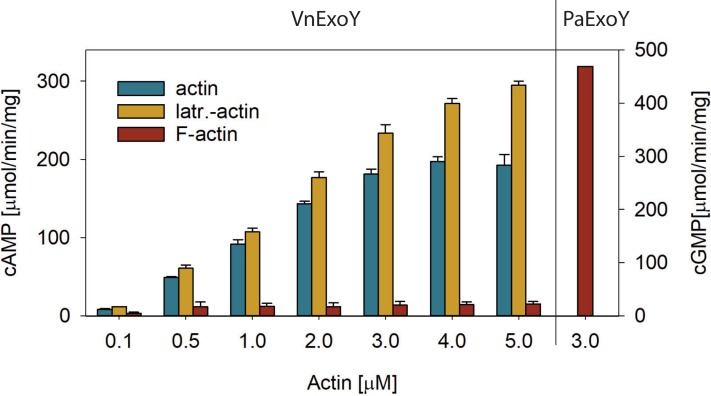
Activation of VnExoY by polymerized or non-polymerized actin *in vitro*. Reactions containing skeletal α-actin polymerized (F-actin) or not (actin) to steady state or prevented from polymerization by the addition of latrunculin A (latr.-actin). Latrunculin A was present at a twofold excess over actin and was preincubated with G-actin for 10 min at room temperature before conversion to Mg-ATP-actin. Actin and VnExoY (5 ng) were preincubated for 10 min at 30°C before the addition of substrate (2 mM ATP) to start the reaction and allowed to proceed for 30 min. Functionality of F-actin was tested in PaExoY-catalyzed cGMP synthesis reactions. Error bars correspond to s. d. of two independent experiments.

*P*. *aeruginosa* ExoY shows preferential activation by F-actin over G-actin [6]. In order to test whether the same is true for VnExoY, we measured activity of VnExoY in the presence of pre-formed actin filaments (steady-state polymerized F-actin) or G-actin that was free to polymerize during the activity assay. Fig 1 shows that the activation of VnExoY was largely diminished when preformed F-actin was used. We then tested whether the addition of latrunculin A, a small macrolide inhibitor of actin polymerization, could affect the activation of VnExoY. As shown in Fig 1, latrunculin A did not diminish activation of VnExoY in contrast to what was seen with PaExoY [6]. Polymerization of actin is therefore not required for the activation of VnExoY and rather seems to strongly interfere with it.

**Fig 2:**
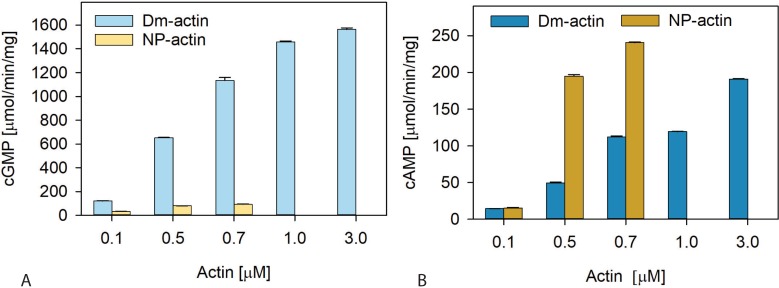
Non-polymerizable mutant actin efficiently activates VnExoY but is impaired in the activation of PaExoY. Non-polymerizable actin from *D*. *melanogaster* (NP-actin at 0.1, 0.5 or 0.7 μM) or the corresponding wild-type control (Dm-actin at 0.1, 0.5, 0.7, 1.0 or 3.0 μM) were used to activate 1 ng PaExoY **(A)** or 10 ng VnExoY **(B)**. Reactions were performed in reaction buffer containing 50 mM KCl and started by the addition of 2 mM substrate (ATP or GTP) and incubated for 30 or 10 min at 30°C for VnExoY or PaExoY, respectively. Error bars correspond to s. d. of two independent experiments.

To verify the inhibiting effect of actin polymerization on the activation of VnExoY, we used a non-polymerizable mutant actin, derived from *Drosophila melanogaster* cytoplasmic actin (here named: NP-actin), which has lost the ability to polymerize due to the presence of 2 substitutions at amino acid residues 205 and 244 (A205E and P244K) [10]. The recombinant mutant NP-actin and the corresponding wild-type protein (Dm-actin) were produced in SF9 insect cells and purified to homogeneity [11]. We then compared the ability of the wild-type and non-polymerizable mutant protein to activate PaExoY and VnExoY. The results shown in Fig 2 confirm our assumption that polymerization affects the 2 enzymes in an opposite way: whereas the mutant NP-actin is largely unable to activate PaExoY, it is an efficient activator of VnExoY, even better than the wild-type actin from *D*. *melanogaster*.

**Fig 3:**
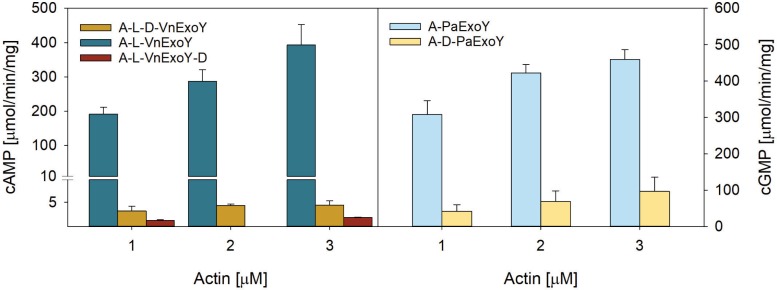
Inhibitory effect of the protein DNase I on the activation of VnExoY and PaExoY by actin. Synthesis of cAMP or cGMP was catalyzed by 5 ng VnExoY or PaExoY activated by Mg-ATP-actin (skeletal muscle α-actin) in the presence or absence of DNase I in twofold excess. Reactions with VnExoY were performed in the presence of latrunculin A. Reactions were started by the addition of 2 mM substrate (ATP or GTP) and incubated for 15 min or 30 min at 30°C. A: skeletal muscle α-actin; L: latrunculin A; D: DNaseI. The order of addition of the reagents in the reaction mixture is indicated in the legend: for example, A-L-D-VnExoY indicates that actin (A) was first incubated with latrunculin (L), followed by the addition of DNase I (D), followed by the addition of VnExoY. Each addition of reagents was followed by a 10 min incubation before adding the next one. All pre-incubations were performed at room temperature before the addition of the enzyme and at 30°C after the addition of enzyme. Ca-ATP-actin was converted to Mg-ATP actin after the addition of latrunculin A for A-L reactions or DNase I for A-L-D or A-D. Error bars correspond to s. d. of at least two independent experiments.

We also examined the effect of DNase I on actin activation of PaExoY or VnExoY. DNase I binds between subdomain 2 and 4 of G-actin to form a tight complex with G-actin with a dissociation constant (Kd) in the nM range [12] and prevents its polymerization. Fig 3 shows that DNase I strongly interfered with the activation of both AA-NC enzymes. Inhibition of PaExoY by DNase I is likely due to the blockage of actin polymerization, which is mandatory for PaExoY activation. In contrast, the inhibitory effect of DNase I on the activation of VnExoY suggests a potential overlap between the binding site(s) of DNase I and VnExoY on actin monomers. As shown in Fig 3, this inhibitory effect was independent of the order of addition of reagents as it was also observed when actin and VnExoY were preincubated before adding DNase I.

### VnExoY interacts with G-actin

To further demonstrate binding of VnExoY to G-actin, we performed fluorescence detected sedimentation velocity experiments using analytical ultracentrifugation (AU-FDS) with latrunculin-stabilized G-actin labeled with the fluorescent dye Alexa 488. The sedimentation coefficient (s) of labeled actin was determined alone and in the presence of varying concentrations of unlabeled VnExoY.

**Fig 4:**
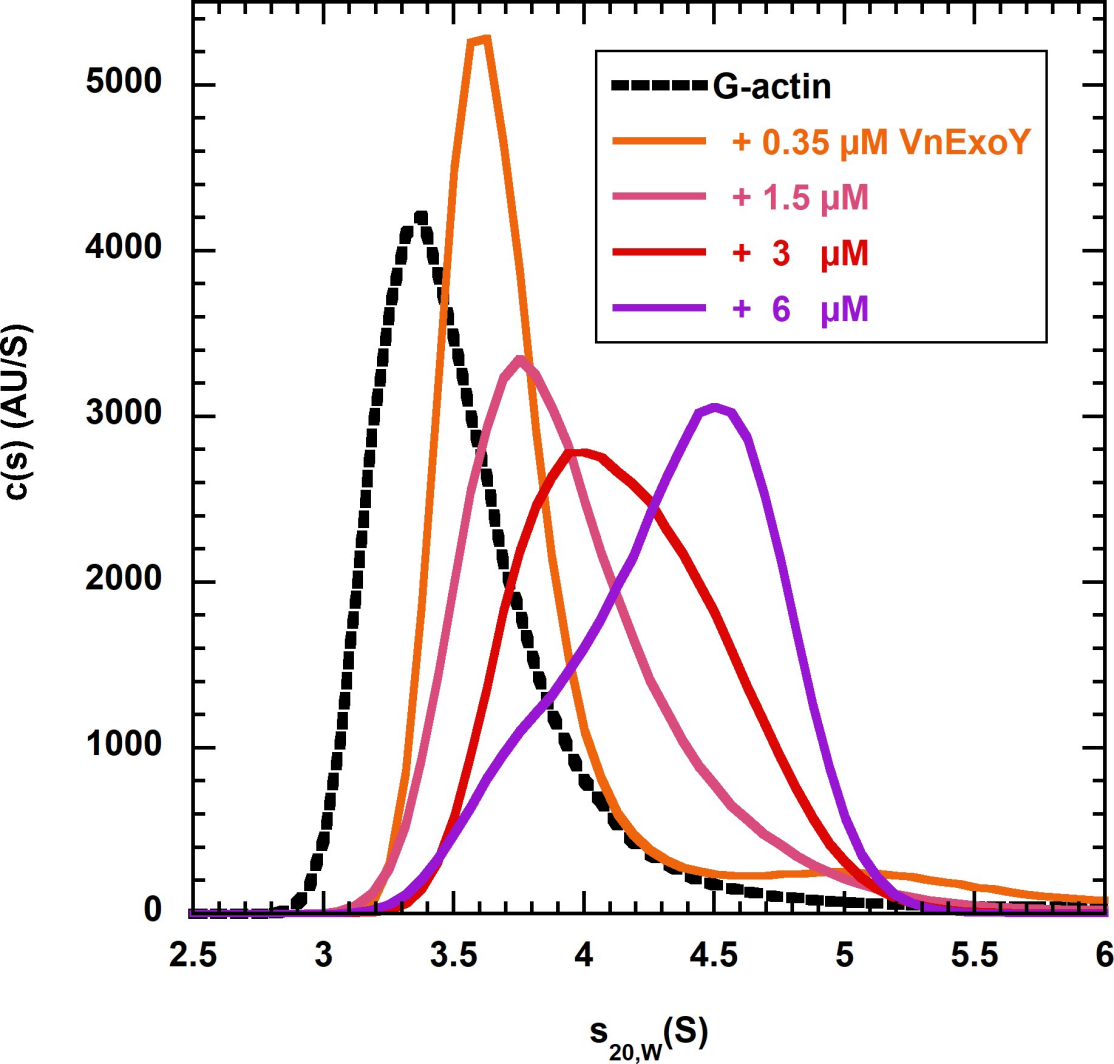
Sedimentation velocity analysis of labeled G-actin alone or with unlabeled VnExoY. Sedimentation coefficient distribution c(s) (s_20,w_ values) determined from analysis of the mixture of 60 nM Alexa 488 labeled G-actin (0.3 μM total, 20% Alexa 488 labeled, 2.5 μM Latrunculin A) with 0 (black dashed line), 0.35 (orange), 1.5 (pink), 3 (red) or 6 (violet) μM unlabeled VnExoY by fluorescence-detected sedimentation velocity.

With free labeled G-actin (0.3 μM, 20% Alexa 488 labeled, 42.5 kDa) a peak with an s_20,w_ value of 3.4 S was observed in the analysis of actin sedimentation coefficient distribution (Fig 4), corresponding to a globular actin monomer. Upon addition of VnExoY (incremental concentrations from 0.35 to 6 μM), the sedimentation coefficient distribution of labeled actin shifted to higher s-values (from 3.6 up to 4.5 S), demonstrating the formation of larger species and the interaction of VnExoY with actin. The peak position and shape were concentration dependent and the relative proportion of the larger species in the peak increased with increasing concentrations of VnExoY. The strong concentration-dependence of the peak position indicated that G-actin and VnExoY form a complex in rapid reversible equilibrium. Because of this rapidly reversible interaction the AU-FDS data did not allow to accurately determine the stoichiometry of the G-actin:VnExoY interaction.

### VnExoY does not interact with F-actin

**Fig 5:**
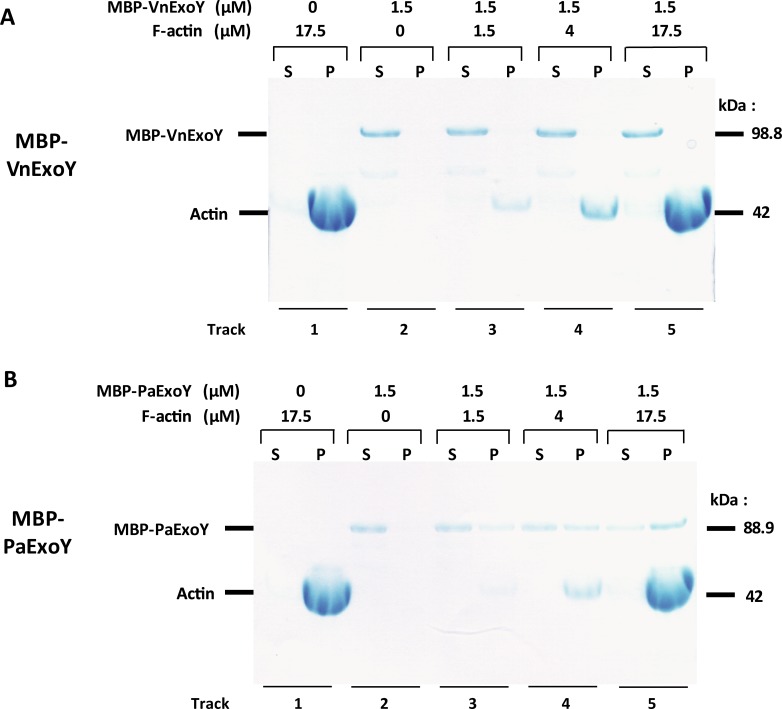
Cosedimentation assays show interaction between F-actin and MBP-PaExoY but not MBP-VnExoY. MBP-VnExoY **(A)** or MBP-PaExoY **(B)** at 1.5 μM were cosedimented at 200,000 g alone or with the indicated concentrations of Mg-ATP-actin (skeletal muscle α-actin), beforehand polymerized to steady state (as shown by control lanes 1 without the toxins). Supernatant (S) and pellet (P) fractions were separated by SDS-PAGE and stained by Coomassie blue.

The preferential activation of VnExoY by G-actin as compared to polymerized actin suggested that this enzyme does not interact with F-actin. We performed cosedimentation assays with increasing amounts of F-actin (1.5, 4, and 17.5 μM) that was polymerized to steady state keeping the amount of *Vibrio* or *Pseudomonas* nucleotidyl cyclases constant at 1.5 μM. We used toxins fused to the maltose-binding protein (MBP) in these experiments to ensure a better separation from actin by SDS-PAGE. Fig 5A shows that MBP-VnExoY does not cosediment with F-actin and remains in the supernatant at all actin concentrations tested. In contrast, MBP-PaExoY was found predominantly (>70%) in the pellet fraction in samples containing 17.5 μM F-actin (Fig 5B). The lack of interaction between polymerized actin and VnExoY is in agreement with the negligible activation of this enzyme by F-actin.

### Activation of VnExoY by different isoforms

**Fig 6:**
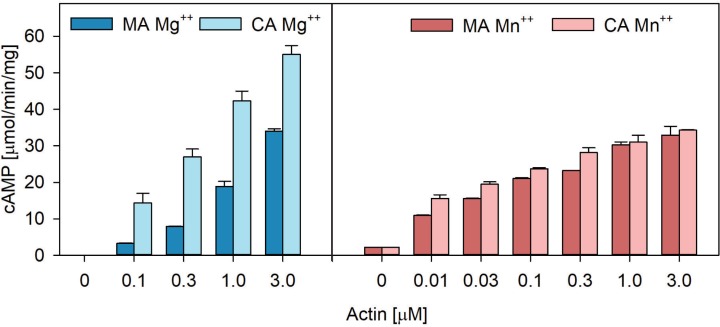
Activation of VnExoY-catalyzed cAMP synthesis by different mammalian isoforms of actin. We compared VnExoY activity in the presence of different mammalian actin isoforms. VnExoY at 3.7 nM (10 ng) was activated by non-muscle actin (CA, a mixture of 85% β- and 15% γ-actin), or muscle α-actin (MA) at the indicated concentrations in the presence of 15 mM Mg^2+^ (left) or Mn^2+^ (right). Reactions were started by the addition of ATP after a 10 min preincubation of VnExoY and actin at 30°C and were continued for 30 min. Error bars correspond to s.d. of two independent experiments.

We then compared activation of VnExoY by different mammalian actin isoforms: the skeletal muscle actin (MA) from rabbit (α-actin) and the cytoplasmic actin (CA) from human platelets (consisting of 85% β- and 15% γ-actin). Actins were kept monomeric by the addition of latrunculin A and enzymatic assays were performed in the presence of either Mg^2+^ or Mn^2+^. As seen in Fig 6, both actins efficiently activated VnExoY. However, while VnExoY was similarly activated by the two preparations of actin in the presence of Mn^2+^ (right panel), in the presence of Mg^2+^, VnExoY was somewhat more efficiently activated by cytoplasmic actin than by muscle actin (with up to 2 folds difference in maximal activities, left panel). These mammalian actin isoforms are, however, highly homologous with sequence similarity above 93.6% (S1 Fig). Further structural and biochemical investigations will be required to understand in detail the basis for these specificities. Moreover, in the presence of Mn^2+^, VnExoY appears to be substantially activated by very low concentrations of actin (e.g. 10 nM, both cytoplasmic or muscle actin), while in the presence of Mg^2+^, similar activation of VnExoY required at least 10-fold more cytoplasmic actin. Interestingly, the background activity, measured in the complete absence of actin, was also considerably higher in buffer containing Mn^2+^ (2 μmol/min/mg) as compared to that measured in the presence of Mg^2+^ (about 0.01 μmol/min/mg). This suggests that Mn^2+^ might induce conformational changes leading to a partially active enzyme.

### Substrate specificity

**Fig 7:**
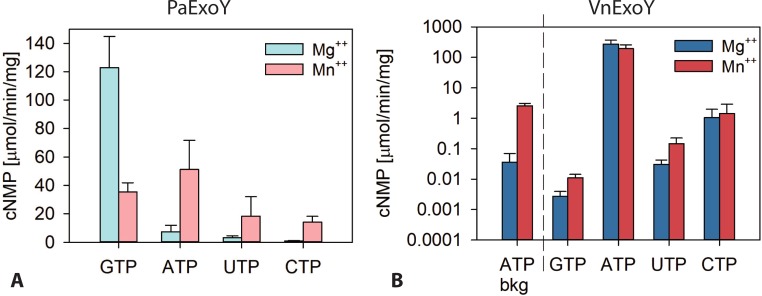
**Substrate specificity of (A) PaExoY and (B) VnExoY. (A)** Reactions containing 5 ng or 10 ng (for GTP) or 50 or 100 ng PaExoY (for ATP, UTP, CTP) that was activated by Mg-ATP-actin (3 μM skeletal α-actin) were started by the addition of NTP substrate (2 mM) and allowed to proceed for 15 or 30 min. Mg^2+^ or Mn^2+^ was present at 15 mM. **(B)** Reactions were performed in reaction buffer containing 50 mM KCl and contained 5 ng or 10 ng (for ATP), 5 μg or 10 μg (for GTP or UTP), 0.25, or 0.5 μg (for CTP) of VnExoY that was activated by Mg-ATP-actin (3 μM skeletal α-actin) or in the absence of actin (ATP bkg) were started by the addition of NTP substrate (2 mM) and allowed to proceed for 10 or 20 min (ATP), 60 or 120 min (GTP), or 30 or 60 min (UTP, CTP). Values were extracted from the linear slope of conversion to cNMP or correspond to averages of specific activities measured in reactions containing different amounts of enzyme. Error bars correspond to s.d. of at least two independent experiments.

PaExoY has been shown to have a broad substrate specificity. This was established by measuring accumulation of cNMPs in cells infected by *P*. *aeruginosa* or in cells transfected with plasmids expressing PaExoY [13, 14]. In agreement with these studies, we previously reported that GTP is a strongly preferred substrate, as compared to ATP, when we measured activities of the purified toxin *in vitro* and in the presence of F-actin. On the other hand, GTP was not a substrate for VnExoY when measured *in vitro* in the presence of actin [6]. Similarly, the ExoY-like MARTX module from *V*. *vulnificus*, a close homologue of VnExoY, does not synthesize cGMP *in vitro* in the presence of eukaryotic cell extract of CHO cells [8]. To explore in more detail the potential differences in substrate specificity between PaExoY and VnExoY, we measured activities of both enzymes in the presence of different nucleotides: ATP, GTP, CTP, and UTP. Fig 7A shows substrate preference of PaExoY in the presence of MgCl_2_ or MnCl_2_. While the guanylate cyclase activity of PaExoY is lower in the presence of Mn^2+^, the catalytic activity to synthesize cAMP, cUMP and cCMP is increased under these conditions. The substrate specificity of the F-actin activated PaExoY can be summarized as follows: GTP>ATP≥UTP>CTP in the presence of Mg^2+^ and ATP = GTP>UTP = CTP in the presence of Mn^2+^. Importantly, the enzymatic activities with the non-canonical substrate UTP and CTP measured *in vitro* with PaExoY clearly did not match the intracellular accumulations of cNMPs measured in cells infected with ExoY-expressing *P*. *aeruginosa* or transfected with plasmids expressing ExoY [13]. In these conditions, cUMP was shown to accumulate to levels equal to, or even exceeding that of cGMP, while in our *in vitro* assays UTP and CTP were only poor substrate in the presence of Mg^2+^. The discrepancy between *in vitro* and *in vivo* data may be explained by an inefficient degradation of cUMP or cCMP by phosphodiesterases (PDEs) *in vivo* as compared to that of cAMP and cGMP. This would result in a quantitative accumulation of the unconventional cyclic nucleotides over time in infected cells. Among the PDEs that were tested for their ability to hydrolyze cUMP, PDE3A, 3B and 9 were found to be active on this substrate in addition to their activity on cAMP and/or cGMP [15]. PDE expression levels, availability due to compartmentalization and potentially the competition with other cNMP substrates could thus contribute to the regulation of the particular cNMP levels in cells. In addition, the level of synthesis of different cNMPs could be affected by the substrate availability of a particular NTP. Peculiarly, cUMP was the only cNMP that could be detected in a recent *in vivo* study in lung tissue or serum of mice infected with *P*. *aeruginosa* depending on the presence of ExoY expressed from a multicopy number plasmid. cUMP was not detected, however, under more physiological conditions namely when *P*. *aeruginosa* strains carrying a single chromosomal were used to infect mice [16].

The substrate specificity of VnExoY (Fig 7B) is much more restricted than that of PaExoY. Catalytic activities with GTP as substrate are very close to the detection limit of 0.001 μmol/min/mg. This is in agreement with previous results indicating that GTP is not a substrate for VnExoY [6, 8]. Catalytic activities with CTP and UTP can be detected but are less than 1% of that measured with ATP. Substrate specificity of VnExoY can be summarized as follows: ATP>>>CTP>UTP.

The observed differences in substrate specificity depending on the presence of Mn^2+^ or Mg^2+^ were more prominent with PaExoY as compared to VnExoY. Similar effects were also reported for other nucleotidyl cyclases such as soluble guanylyl cyclase α1β1, EF from *B*. *anthracis* and CyaA from *B*. *pertussis* [17, 18], which also showed a preferential utilization of non-canonical substrates in the presence of Mn^2+^ as compared to Mg^2+^. Due to its higher concentrations in cells, Mg^2+^ is more likely to be used under physiological conditions. Future experiments might reveal the physiological relevance of using Mn^2+^in cells and potentially specific conditions under which this ion is used instead of Mg^2+^.

### Mutants affecting the catalytic activity of PaExoY or VnExoY

Earlier studies have shown that PaExoY variants harboring the K81M or K88I substitutions have drastically reduced catalytic activity [19]. These variants are commonly used as negative control for *in vivo* assays of ExoY biological activity or toxicity [20, 21]. To assess the toxicity of these variants in a yeast model, the wild-type or mutant PaExoY were expressed in *S*. *cerevisiae* under the control of an inducible promoter and effects on growth were observed (Table 1 and S2 Fig). Strikingly, we found that both mutants displayed considerable toxicity in yeast despite the fact that the purified variants exhibit very low enzymatic activity when assayed *in vitro* in the presence of actin. Combination of both mutations K81M and K88I was needed to fully abolish PaExoY toxicity in yeast and completely eliminate enzymatic activity *in vitro*. The equivalent mutations K117M and/or K124I were introduced into VnExoY (according to sequence alignment, see S3 Fig) and the *in vitro* activity and *in vivo* toxicity in yeast of the corresponding variants were tested (Table 1 and S2 Fig). As above, the double substitution K117M/K124I fully abolished VnExoY toxicity and enzymatic activity. However, in contrast to PaExoY, the single K117M mutant was largely devoid of toxicity in the yeast model although it still exhibited a detectable residual enzymatic activity. Particular cNMPs are likely to induce different toxicity levels. The observed differences in substrate specificity between PaExoY and VnExoY and consequently the accumulation of different cNMPs pools could therefore generate unequal toxicity levels.

**Table 1:** Effects of mutations in PaExoY or VnExoY on toxicity in *S*. *cerevisiae* and *in vitro* activity.

Construct			Glucose (repressed)	Raffinose (background induction)	Galactose (highly induced)	*In vitro* activity [%]
vector		1	++	++	++	n.a.
PaExoY	wt	2	++	+	-	100
	K81M	3	++	++	+	0.01–0. 1
	K88I	4	++	++	-	n.t.
	K81M/K88I	5	++	++	++	n.d.
VnExoY	wt	6	++	+	-	100
	K117M	7	++	++	++	0.01–0. 1
	K124I	8	++	++	-	n.t.
	K117M/K124I	9	++	++	++	0.0005–0.005

*S*. *cerevisiae* MH272-3fα cells carrying a vector control (YEpGal555) or expressing the indicated proteins were grown on minimal agar plates at 30°C. Cell suspensions were normalized to an OD600 of 1.0 and serial dilutions (5-fold) were applied as 3 μl drops on agar plates containing 2% galactose (for high induction of P_*Gal*_), 2% raffinose (background induction levels of P_*Gal*_) or 2% glucose (for repression of P_*Gal*_).

++: unaffected growth comparable to the vector control

+: intermediate growth

-: absence of growth

wt: wild type; n.a.: not applicable; n.d.: non detectable; n.t.: not tested. Original data are shown in S2 Fig.

All together, these data highlight the high sensitivity of *S*. *cerevisiae* to the toxic action of PaExoY and VnExoY. Also, the commonly used K81M or K88I mutants of PaExoY, and the corresponding K117M and K124I mutant of VnExoY have detectable residual catalytic activity *in vitro* as well as *in vivo*. The residual activity in these single-residue changed toxin variants may exert some residual toxic effects when these modified AA-NCs are expressed or delivered to eukaryotic target cells (as clearly evidenced here in yeast) and might explain the effects on cell morphology or intracellular contacts previously observed with the supposedly catalytic inactive form of PaExoY K81M [20, 21]. Therefore, our data strongly suggest that the dual mutants K81M/K88I and K117M/K124I of PaExoY and VnExoY, respectively, should be used as truly enzymatically inactive variants of AA-NC toxins in all *in vivo* assays.

## Conclusions

Here we show that two AA-NCs, VnExoY and PaExoY, despite having actin as common activator in eukaryotic cells differ significantly: while PaExoY requires F-actin for maximum activation, VnExoY is better activated by G-actin. Whereas ExoY represents to our knowledge the only example of a bacterial toxin that is activated by F-actin, G-actin has been shown previously to activate a bacterial toxin secreted by the T3SS namely YopO/YpkA, a multidomain protein from *Yersinia* species (*Y*. *enterocolitica* and *Y*. *pseudotuberculosis*, respectively), which is involved in the disruption of the actin cytoskeleton [22, 23]. G-actin is also a frequent target of bacterial toxins, which can affect the polymerization state of actin in different ways by introducing modifications, such as ADP-ribosylation at different position or crosslinking (for reviews see refs.[24,25]).

Interaction with either F- or G-actin could in turn affect the localization of the enzyme within cells and thus the localization of the generated cNMPs. Future investigations will reveal whether the interaction of AA-NC toxins with either F- or G-actin can also induce different alterations of the actin cytoskeleton dynamics in host cells independently from their catalytic activity [6, 26].

In addition, the observed differences in substrate specificity indicate that these enzymes might target different pathways. While the F-actin activated PaExoY can generate both canonical purine and non-canonical pyrimidine cNMPs *in vitro* (Fig 6) or *in vivo* [13, 27], the VnExoY is a rather selective adenylate cyclase enzyme similar to the well-known calmodulin-activated adenylate cyclase toxins EF from *B*. *anthracis* and CyaA from *B*. *pertussis* [13, 28].

The observed differences within the AA-NCs concerning their activation and substrate specificity indicate that their mode of action in pathogenicity could differ considerably. The characterization of additional ExoY-like proteins could clarify whether AA-NCs fall into 2 subgroups and uncover what determines interaction with either G- or F-actin on one hand and substrate specificity on the other hand.

## Materials and methods

### Strains, plasmids and growth conditions

Strains, plasmids and primers are described in S1 Table.

*E*. *coli* strains were grown in lysogeny broth (LB). Ampicillin (100 μg/ml) was added for plasmid maintenance in *E*. *coli*. *S*. *cerevisiae* strains were grown in minimal medium containing yeast nitrogene base without amino acids (Difco) containing galactose (SG), glucose (SD) or raffinose (SR) supplemented if required with uracil, histidine, leucine, tryptophan or adenine. Glucose, galactose or raffinose were present at 2%. *S*. *cerevisiae* strains were transformed using the lithium-acetate method [29].

Plasmids p1648 and pB14 for the expression of wild-type VnExoY and VnExoY^K117M^ in *S*. *cerevisiae* were constructed as follows: Primers 1280 and 40 or b16 and b17 were used to PCR-amplify the according fragments from pUM522 or pUM530, respectively. The XhoI, KpnI digested fragments were cloned into YEpGAL555.

Plasmids pB15 and pB16 for the expression of VnExoY^K124I^ and VnExoY^K117M/K124I^: Mutations were introduced by PCR-based site-directed mutagenesis [30] with primers b18 and b19 and template p1648 or b18 and b19 and template pB14, respectively. The XhoI, KpnI digested fragments were inserted into YEpGAL555.

For the construction of p1594 expressing PaExoY^K81M^, the PCR-amplified fragment (primers 1259 and 1260 and pUM498 at template), was digested with XhoI and KpnI and inserted into YEpGAL555.

Plasmids pB46 and p1682 expressing PaExoY^K88I^ or PaExoY^K81M/K88I^ were constructed by PCR-based site-directed mutagenesis [30] using primers b61, b62 and template p1593 or primers 1307, 1308 and template p1594, respectively. XhoI, KpnI digested fragments were inserted back into YEpGAL555.

Plasmid pUM530 expressing VnExoY^K116M^ in pBAD33 was created as follows: the mutation was introduced by PCR using primers UM364 and UM365 on pUM522, TA-subcloning into pGEMT-Easy (Promega). The SalI, BglII fragment containing the mutation was used to replace the Sal, BglII fragment from pUM524 (VnExoY in pBAD33).

To generate pUM533 expressing VnExoY^K117M^ from lambda P_*L*_ controlled by the temperature sensitive cI (cI857), the MluI, EcoRI fragment of pUM522 was replaced with that of pUM530.

Plasmid pUM536 (expressing VnExoY^K117M/K124I^ from lambda P_*L*_ controlled by the temperature sensitive cI), was constructed by replacing the MluI, EcoRI fragment of pUM522 by that from pB16.

Plasmid pEA11 for the expression of MBP-PaExoY was previously described [6]. pLR152 for the expression of MBP-VnExoY was constructed as follows: Primers o59 and o60 were used to PCR-amplify the fragment from pUM522. The BamHI, XhoI digested fragment was cloned into pEA11.

### Purification of PaExoY, VnExoY and actin

Recombinant PaExoY containing a C-terminal Flag-His-Tag was purified by nickel affinity chromatography under denaturing conditions as described before [6] and kept in 500 mM NaCl, 20 mM Tris, pH 9.0, 1 mM DTT (1,4-Dithiothreitol), 10% glycerol.

VnExoY was purified under native conditions from the soluble protein fraction and kept in 150 mM NaCl, 20 mM Tris pH 8.0, 1 mM DTT, 10% glycerol. All experiments were reproduced with several distinct enzyme preparations of both PaExoY and VnExoY and showed highly consistent results in terms of relative substrate specificities and activation by F versus G actin.

The fusion constructs of PaExoY and VnExoY with an N-terminal maltose-binding protein (MBP, 40.4 kDa), designed as follows: (His-Tag)-(MBP)-(PreScission-site)-(PaExoY/VnExoY)-(Strep-tagII) and referred in the text as MBP-PaExoY or MBP-VnExoY were purified under non-denaturing conditions successively from HisTrap, StrepTrap, and Superdex 200 16/60 columns using standard protocols. For analytical ultracentrifugation experiments, VnExoY was cleaved from MBP-VnExoY using PreScission protease (GE27-0843-01, Sigma Aldrich) before the Strep Trap purification step. Proteins were stored in 15 mM Tris pH 8.0, 75–150 mM KCl, 1 mM DTT. Activity assays to verify the functionality of the MBP-fusion proteins showed similar specific activities as the corresponding PaExoY or VnExoY enzymes for the synthesis of cGMP or cAMP, respectively.

Commercial DNase I (10104159001, Roche) was further purified on a size-exclusion chromatography column (Superdex 75 16/60, buffer: 0.1 M KCl, 15 mM Hepes pH 7.5, 5 mM CaCl_2_, with protease inhibitors) to remove protease contamination present in the commercial DNase reagent.

α-actin (UniProt P68135) was prepared from acetone dried powder derived from Oryctolagus cuniculus (Rabbit) back and leg muscles in our laboratory according to the method of Spudich and Watt [31] as described previously [6].

*D*. *melanogaster* cytoplasmic wild-type (Dm-actin, UNIPROT accession number: ACT1_DROME) and mutant actin (actin NP) were produced and purified from SF9 insect cells infected with recombinant Baculoviruses as described before [11].

Pure non-muscle actin (CA) from human platelets (Reference APHL99, a mixture of 85% β- and 15% γ-actin) was obtained from Cytoskeleton, Inc.

### Interaction between PaExoY/VnExoY and actin using Analytical Ultracentrifugation (AUC)

Fluorescence-detected sedimentation velocity (AU-FDS for Analytical Ultracentrifuge Fluorescence Detection System) experiments were conducted with a Beckman XL-I (Beckman-coulter, Palo Alto, USA) analytical ultracentrifuge using an An-50Ti rotor equipped with Aviv fluorescence detection system (AU-FDS, Aviv Biomedical). Actin was fluorescently labeled with Alexa-488 N-hydroxysuccinimide (NHS) ester (A20000, Thermofischer Scientific) as previously described [32]. In brief, functional Alexa 488-actin was prepared by labeling the surface amine residues of polymerized F-actin in (20 mM PIPES pH 6.9, 0.2 mM CaCl_2_, 0.2 mM ATP, 0.1 mM KCl). After labeling, F-actin was pelleted (at 300,000 g for 40 min at 20°C), and subjected to two cycles of depolymerization, polymerization, and pelleting to ensure removal of free dye and that labeled actin can properly assemble. Depolymerized labeled G-actin was stored on ice in G-buffer in the dark. The fraction of labeled actin was 20%.

VnExoY, purified in native conditions and cleaved from MBP-VnExoY, was used for the AUC experiments. The buffer used in AUC experiments was (100 mM KCl, 15 mM Hepes pH 7.5, 3 mM ATP, 5 mM CaCl_2_, 1 mM MgCl_2_, 0.5 mM cAMP, 0.5 mM pyrophosphate, 2.5 μM Latrunculin A, 0.23% DMSO, 4% Glycerol, 0.55 mg/ml BSA (8μM)) for all other experiments. Samples and buffers were centrifuged at 15,000 g for 10 min prior to AUC experiments and measuring solvent properties. Alexa 488 labeled G-actin at a final concentration of 60 nM was loaded into Epon charcoal-filled two-sector 12 mm path-length cells. Samples were centrifuged at 40,000 rpm (116 369 g) and 20°C. Fluorescence data were obtained at an excitation wavelength of 488 nm and emission wavelengths between 505 and 565 nm, scanning all cells simultaneously every 6 min. Sedimentation velocity data were analyzed using SEDFIT software [33] and the partial specific volume used for Alexa 488-G-actin was 0.723 ml/g. Buffer viscosities and densities were experimentally measured with an Anton Paar microviscosimeter (Lovis 2000) and density meter (DMA 4500), respectively.

### Cosedimentation assays

For cosedimentation assays we used MBP-VnExoY or MBP-PaExoY and rabbit skeletal muscle α-actin (Fig 4). The fusion of the maltose-binding protein (MBP) to the N-terminal of PaExoY did not influence its interaction with F-actin [6]. MBP-VnExoY (98.8 kDa) and MBP-PaExoY (88.9 kDa) allowed separating and quantifying unambiguously by densitometry the fraction of the bound toxin from actin at 42 kDa by SDS-PAGE, while VnExoY (M.W. of 53.3 kDa) and PaExoY (43 kDa) were migrating too close to actin. 1.5 μM of MBP-VnExoY/-PaExoY were incubated in (50 mM Tris pH 7.8, 50 mM KCl, 2 mM MgCl_2_, 1 mM DTT, 4 mM ATP, 0.5 mM GTP, 3 mM cAMP) for 1h with increasing amounts of F-actin (0, 1.5, 4 and 17.5 μM) polymerized overnight to steady state in the same buffer. The supernatant/unpolymerized (S) and pellet/polymerized (P) fractions were separated by an ultracentrifugation for 30 min at 200,000 g, resolved by 10% SDS-PAGE and detected by coomassie blue staining. The ExoY-bound fraction was quantified by densitometry using the ImageJ software. A control of 17.5 μM F-actin alone (without MBP-VnExoY/-PaExoY) is shown in lane 1 showing that actin is mostly in the pellet fraction after polymerization.

### Quantification of cAMP or cGMP synthesis *in vitro*

PaExoY-catalyzed synthesis of cAMP and cGMP synthesis were measured in 50 μl reactions as described previously [6] containing 50 mM Tris pH 8.0, 0.5 mg/ml BSA, 200 mM NaCl, 1 mM DTT, MgCl_2_ or MnCl_2_ as indicated, 2 mM ATP or GTP spiked with 0.1 μCi of [α-^33^P] ATP or [α-^33^P] GTP, respectively, PaExoY and indicated amounts of purified actin. cUMP and cCMP synthesis was measured similarly but using α-^32^P labeled UTP and CTP substrates. Reaction conditions for VnExoY were similar except that NaCl was replaced by 50 mM KCl when indicated.

Mg-ATP-actin was prepared from Ca-ATP-actin for measurements of PaExoY activities as described before [6].

Latrunculin A was purchased from tebu-bio (produced by Focus Biomolecules), added to G-actin in 2-fold access, and incubated for 10 min at room temperature before use in activity assays.

## Supporting information

S1 Fig Multiple sequence alignment and sequence similarity between the different actin isoforms used in VnExoY activity assays. (RTF)

S2 Fig Effects of mutations in PaExoY or VnExoY on toxicity in *S*. *cerevisiae*. (DOCX)

S3 Fig Sequence alignments of MARTX ExoY-Like domains from *Vibrio nigripulchritudo* (Vn-ExoY-MARTXdo, from UniProt F0V1C5_9VIBR, used in the current study), *Vibrio vulnificus* (Vn-ExoY-MARTXdo, from UniProt A0A023NA98_VIBVL), *Vibrio cholerae* (Vc-ExoY-MARTXdo, from UniProt D7H8T5_VIBCL), with ExoY proteins from *Pseudomonas aeruginosa* strain UCBPP-PA14 (Pa-ExoY-PA14-st, UniProt A0A0H2ZM07_PSEAB), and *Pseudomonas aeruginosa* strain PAO1 (Pa-ExoY-PAO1-st, UniProt Q9I1S4_PSEAE, used in the current study). (RTF)

S1 Table Strains, plasmid, and primers. (DOCX)
