# Supplementary material for: Differential regulation of actin-activated nucleotidyl cyclase virulence factors by filamentous and globular actin
Source: PLoS One. 2018 Nov 12;13(11):e0206133. doi: 10.1371/journal.pone.0206133 (PMC6231621; doi:10.1371/journal.pone.0206133)
Supplement: S1 Fig — (RTF) [file pone.0206133.s001.rtf]

A
                                            D25N mutation in ACT_YEAST (S. cerevisiae)
                                            ▼    
c_ACT1_DROME    1 MC-DEEVAALVVDNGSGMCKAGFAGDDAPRAVFPSIVGRPRHQGVMVGMGQKDSYVGDEA
c_ACTB_HUMAN    1 M--DDDIAALVVDNGSGMCKAGFAGDDAPRAVFPSIVGRPRHQGVMVGMGQKDSYVGDEA
c_ACTG_HUMAN    1 M--EEEIAALVIDNGSGMCKAGFAGDDAPRAVFPSIVGRPRHQGVMVGMGQKDSYVGDEA
m_ACTS_RABIT    1 MCDEDETTALVCDNGSGLVKAGFAGDDAPRAVFPSIVGRPRHQGVMVGMGQKDSYVGDEA
                    |   |    |      |       |
m_ACTS_RABIT:       3*  7*8* 12     19      27*

c_ACT1_DROME   60 QSKRGILTLKYPIEHGIVTNWDDMEKIWHHTFYNELRVAPEEHPVLLTEAPLNPKANREK
c_ACTB_HUMAN   59 QSKRGILTLKYPIEHGIVTNWDDMEKIWHHTFYNELRVAPEEHPVLLTEAPLNPKANREK
c_ACTG_HUMAN   59 QSKRGILTLKYPIEHGIVTNWDDMEKIWHHTFYNELRVAPEEHPVLLTEAPLNPKANREK
m_ACTS_RABIT   61 QSKRGILTLKYPIEHGIITNWDDMEKIWHHTFYNELRVAPEEHPTLLTEAPLNPKANREK
                                                              |
m_ACTS_RABIT:                                                 105

c_ACT1_DROME  120 MTQIMFETFNTPAMYVAIQAVLSLYASGRTTGIVLDSGDGVSHTVPIYEGYALPHAILRL
c_ACTB_HUMAN  119 MTQIMFETFNTPAMYVAIQAVLSLYASGRTTGIVMDSGDGVTHTVPIYEGYALPHAILRL
c_ACTG_HUMAN  119 MTQIMFETFNTPAMYVAIQAVLSLYASGRTTGIVMDSGDGVTHTVPIYEGYALPHAILRL
m_ACTS_RABIT  121 MTQIMFETFNVPAMYVAIQAVLSLYASGRTTGIVLDSGDGVTHNVPIYEGYALPHAIMRL
                            |                                |
m_ACTS_RABIT:               131                              164

c_ACT1_DROME  180 DLAGRDLTDYLMKILTERGYSFTTTAEREIVRDIKEKLCYVALDFEQEMATAASSSSLEK
c_ACTB_HUMAN  179 DLAGRDLTDYLMKILTERGYSFTTTAEREIVRDIKEKLCYVALDFEQEMATAASSSSLEK
c_ACTG_HUMAN  179 DLAGRDLTDYLMKILTERGYSFTTTAEREIVRDIKEKLCYVALDFEQEMATAASSSSLEK
m_ACTS_RABIT  181 DLAGRDLTDYLMKILTERGYSFVTTAEREIVRDIKEKLCYVALDFENEMATAASSSSLEK
                                        |
m_ACTS_RABIT:                           203*

c_ACT1_DROME  240 SYELPDGQVITIGNERFRCPEALFQPSFLGMEACGIHETTYNSIMKCDVDIRKDLYANTV
c_ACTB_HUMAN  239 SYELPDGQVITIGNERFRCPEALFQPSFLGMESCGIHETTFNSIMKCDVDIRKDLYANTV
c_ACTG_HUMAN  239 SYELPDGQVITIGNERFRCPEALFQPSFLGMESCGIHETTFNSIMKCDVDIRKDLYANTV
m_ACTS_RABIT  241 SYELPDGQVITIGNERFRCPETLFQPSFIGMESAGIHETTYNSIMKCDIDIRKDLYANNV
                                       |           |                        |
m_ACTS_RABIT:                          262         274*                     299

c_ACT1_DROME  300 LSGGTTMYPGIADRMQKEITALAPSTMKIKIIAPPERKYSVWIGGSILASLSTFQQMWIS
c_ACTB_HUMAN  299 LSGGTTMYPGIADRMQKEITALAPSTMKIKIIAPPERKYSVWIGGSILASLSTFQQMWIS
c_ACTG_HUMAN  299 LSGGTTMYPGIADRMQKEITALAPSTMKIKIIAPPERKYSVWIGGSILASLSTFQQMWIS
m_ACTS_RABIT  301 MSGGTTMYPGIADRMQKEITALAPSTMKIKIIAPPERKYSVWIGGSILASLSTFQQMWIT


c_ACT1_DROME  360 KQEYDESGPSIVHRKCF
c_ACTB_HUMAN  359 KQEYDESGPSIVHRKCF
c_ACTG_HUMAN  359 KQEYDESGPSIVHRKCF
m_ACTS_RABIT  361 KQEYDEAGPSIVHRKCF
                        |    
m_ACTS_RABIT:           367*               


B
% pairwise sequence similarity	c_ACT1_DROME
cytoplasmic Dm-actin-5C	c_ACTB_HUMAN
human cytoplasmic b-actin	c_ACTG_HUMAN
human cytoplasmic g-actin	
c_ACTB_HUMAN
human cytoplasmic b-actin	98.1			
c_ACTG_HUMAN
human cytoplasmic g-actin	98.1	98.9		
m_ACTS_RABIT
Rabbit skelet. muscle a-actin	93.6	93.6	93.9	


S1 Fig. Multiple sequence alignment and sequence similarity between the different actin isoforms used in VnExoY activity assays.
A) Multiple sequence alignment of the different actin isoforms used in VnExoY activity assays. Actin isoforms are: c_ACT1_DROME (referred as Dm-actin in the main text) for Drosophila melanogaster cytoplasmic actin-5C (Uniprot accession number: ACT1_DROME), c_ACTB_HUMAN and c_ACTG_HUMAN for human platelet cytoplasmic b- and g-actin (ACTB_HUMAN and ACTG_HUMAN), respectively (CA in the main text), and m_ACTS_RABIT for rabbit (Oryctolagus cuniculus) skeletal muscle a-actin (MA in the main text). Black or grey highlights show amino acid residues that are identical or with similar hydrophilic or hydrophobic side-chain properties in almost all different sequences, respectively. Residues that exhibit a different hydrophilic or hydrophobic property only in muscle actin (m_ACTS_RABIT) are numbered and additionally marked with a red star (*) when they are solvent exposed in actin structure. The location of the mutation D25N in S. cerevisiae actin (ACT_YEAST) which is sufficient to abolish PaExoY cytotoxicity in yeast cells expressing the toxin (1) is shown in green. 
B) Pairwise sequence similarities (in %) between the different actin isoforms according to the sequence alignment in A. 

Reference 

1. 	Belyy A, Raoux-Barbot D, Saveanu C, Namane A, Ogryzko V, Worpenberg L, et al. Actin activates Pseudomonas aeruginosa ExoY nucleotidyl cyclase toxin and ExoY-like effector domains from MARTX toxins. Nat Commun. 2016;7:13582.
