# Supplementary material for: Differential regulation of actin-activated nucleotidyl cyclase virulence factors by filamentous and globular actin
Source: PLoS One. 2018 Nov 12;13(11):e0206133. doi: 10.1371/journal.pone.0206133 (PMC6231621; doi:10.1371/journal.pone.0206133)
Supplement: S2 Fig — (DOCX) [file pone.0206133.s002.docx]

**
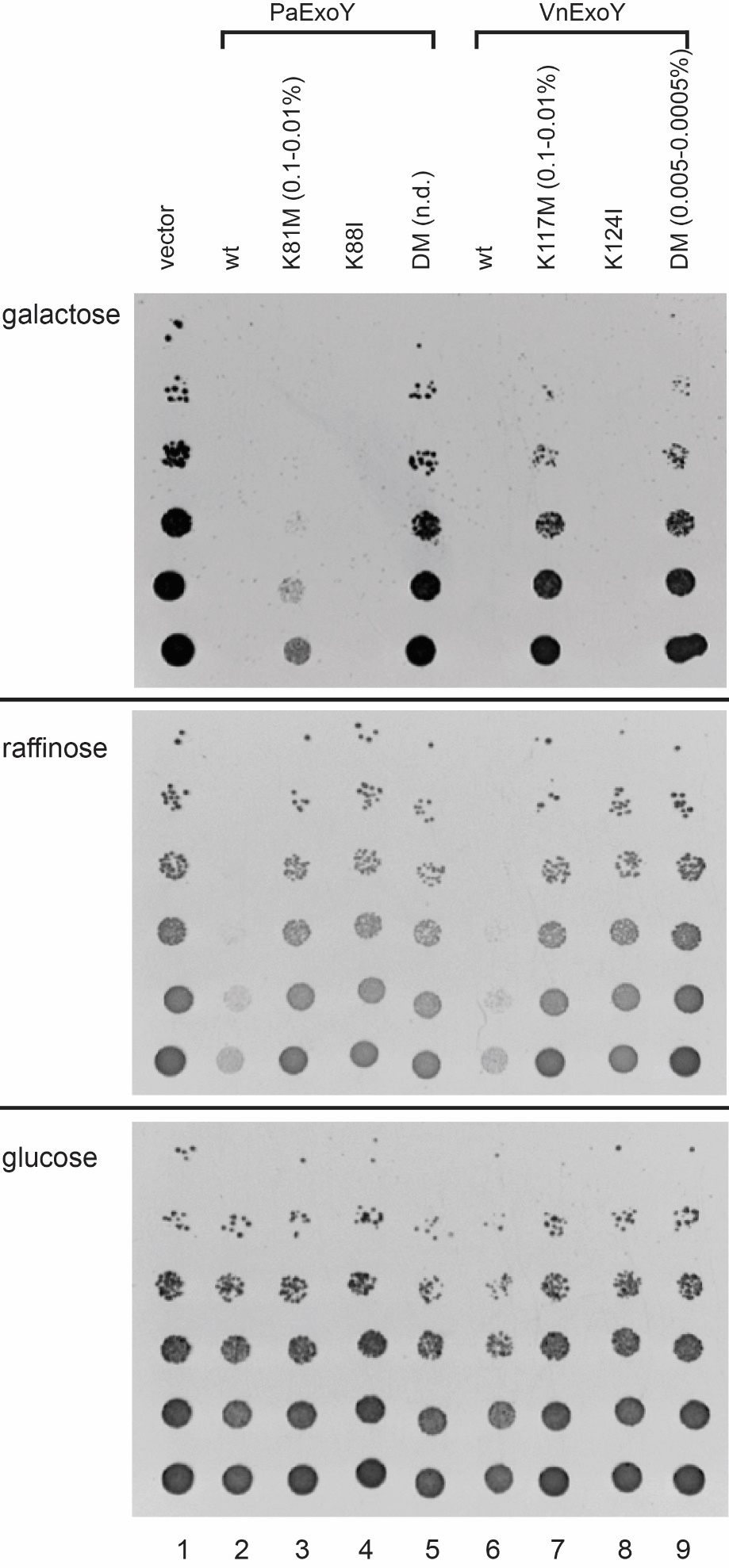
**

**S2 Fig. Effects of mutations in PaExoY or VnExoY on toxicity in *S. cerevisiae*.** *S. cerevisiae* MH272-3fα carrying 1) YEpGal555 (vector), 2) p1593 (PaExoY wt), 3) p1594 (PaExoY^K81M^), 4) pB46 (PaExoY^K88I^), 5) p1682 (PaExoY^K81M/K88I^), 6) p1648 (VnExoY wt), 7) pB14 (VnExoY^K117M^), 8) pB15 (VnExoY^K124I^), or 9) pB16 (VnExoY^K117M/K124I^) were grown on minimal agar plates at 30ºC. Cell suspensions were normalized to an OD600 of 1.0 and 5-fold serial dilutions were applied as 3 µl drops on agar plates. DM: double mutant, wt: wild type. Numbers next to the alleles indicate activities of cGMP or cAMP synthesis in % of wild-type activity for purified PaExoY or VnExoY, respectively. n.d.: non detectable.
