# Supplementary material for: Differential regulation of actin-activated nucleotidyl cyclase virulence factors by filamentous and globular actin
Source: PLoS One. 2018 Nov 12;13(11):e0206133. doi: 10.1371/journal.pone.0206133 (PMC6231621; doi:10.1371/journal.pone.0206133)
Supplement: S3 Fig — (RTF) [file pone.0206133.s003.rtf]

Vn-ExoY-MARTXdo   21 PRQRVTANELQLGDDNAITNAVTSEQEATPNQDGSHKTYQSRDLVLEPIQHPKSIELGMP
Vv-ExoY-MARTXdo   56 KDIRATED---LSVVK----TVASDTELGTNTDAPHKNYQSRDLVLEPIVQPETIELGMP
Vc-ExoY-MARTXdo   57 PTSRRNQA---LTQLKEQITSALLNNETELKIESRPKNYQSRDLVLEPIVQPETIELGMP
Pa-ExoY-PA14-st    1 ---------MRIDGHR----QVVSNATAQPGPLLRPADMQARAL--QDL--FDAQGVGVP

                                                           motif I
                                                VnExoY-K117M      VnExoY-K124I
                                                           |      |
Vn-ExoY-MARTXdo   81 EVDQSVLAEVAERENVIIGVRPVDEKSKSLIASKMYSSKGLFVKAKSSDWGPMSGFIPVD
Vv-ExoY-MARTXdo  109 DSDQKILAEVAERENVIIGVRPVDEKSKSLIDSKLYSSKGLFVKAKSSDWGPMSGFIPVD
Vc-ExoY-MARTXdo  114 DIDQKILAEVAERENVIIGVRPVDEKSKSLIDSKLYSSKGLFVKAKSSDWGPMSGFIPVD
Pa-ExoY-PA14-st   44 VEHALRMQAVARQTNTVFGIRPVERIVTTLIEE-GFPTKGFSVKGKSSNWGPQAGFICVD
Pa-ExoY-PAO1-st   44 VEHALRMQAVARQTNTVFGIRPVERIVTTLIEE-GFPTKGFSVKGKSSNWGPQAGFICVD
                                                           |      |
                                                 PaExoY-K81M      PaExoY-K88I

Vn-ExoY-MARTXdo  141 QSFAKASARR--DLEKFNEYAEQSILSGNAVSANLYLNQVRIEELVSKYESLTPLELDVD
Vv-ExoY-MARTXdo  169 QAFAKASARR--DLDKFNGYAEQSIESGNAVSADLYLNQVRIDELVSKYQSLTALEFDAE
Vc-ExoY-MARTXdo  174 QVFAKASARR--DLDKFNGYAEQSIESGNAVSADLYLNQVRIDELVSKYQSLTALEFDAE
Pa-ExoY-PA14-st  103 QHLSKRENRDTAEIRKLNLAVAKGMDGGAYTQTDLRISQQRLAELVRNFGLVADG-----
Pa-ExoY-PAO1-st  103 QHLSKREDRDTAEIRKLNLAVAKGMDGGAYTQTDLRISRQRLAELVRNFGLVADG-----

                                                                          motif II
Vn-ExoY-MARTXdo  199 SGMYKTTATNGDQTIPFFLNKVTVDDKELWQVHYLREGELAPFKVIGDPVSKQPMTADYD
Vv-ExoY-MARTXdo  227 SGMYKTTATNGDQTVTFFLNKVTVDSKDLWQVHYIKDGKLAPFKVIGDPVSKQPMTADYD
Vc-ExoY-MARTXdo  232 SGMYKTTATNGDQTATFFLNKVTVDSKDLWQVHYMKDGELAPFKVIGDPVSKQPMTADYD
Pa-ExoY-PA14-st  158 VGPVRLLTAQGPSGKRYE-FEARQEPDGLYRIS--RLGRSEAVQVLASPACGLAMTADYD
Pa-ExoY-PAO1-st  158 VGPVRLLTAQGPSGKRYE-FEARQEPDGLYRIS--RLGRSEAVQVLASPACGLAMTADYD

               motif II
Vn-ExoY-MARTXdo  259 LLTVMYTYGDLGPQDKVKQPLTWEQWKESVTYEDLSPKYKARYDNQALYEKQDGASLGMV
Vv-ExoY-MARTXdo  287 LLTVMYSYSDLGPQDKLKQPLTWEQWKESVTYEELTPKYKELYNSEVLYNKKDGASLGVV
Vc-ExoY-MARTXdo  292 LLTVMYSYADLGPQDKVKQPLNWEQWKESVTYEDLTPKYKELYNSEVLYNKKDGASLGVV
Pa-ExoY-PA14-st  215 LFLVAPSIEAHGNGGLDAR------RNTAVRYTPLGAK--DPLSEDGFYGR-EDMARGNI
Pa-ExoY-PAO1-st  215 LFLVAPSIEAHGSGGLDAR------RNTAVRYTPLGAK--DPLSEDGFYGR-EDMARGNI

Vn-ExoY-MARTXdo  319 SDRLKELKDVINTSLGRTDGLEMVHHGADDANPYAVMADNFPATFFVPKHFFDDDGLGEG
Vv-ExoY-MARTXdo  347 SDRLKALKDVINTSLGRTDGLEMVHHGADDANPYAVMADNFPATFFVPKSFFMEDGLGEG
Vc-ExoY-MARTXdo  352 SDRLKALKDVINTSLGRTDGLEMVHHGADDANPYAVMADNFPATFFVPKSFFMEDGLGEG
Pa-ExoY-PA14-st  266 TPRTRQLVDALNDCLGRGEHREMFHHSDDAGNPGSHMGDNFPATFYLPRAME--HRLGEE
Pa-ExoY-PAO1-st  266 TPRTRQLVDALNDCLGRGEHREMFHHSDDAGNPGSHMGDNFPATFYLPRAME--HRVGEE

Vn-ExoY-MARTXdo  379 KGSIQTYFNVNEQGAVVIQNPQEFSNFQQVAINASYRASLNDKWNSGLDSPLFTTKRKLS
Vv-ExoY-MARTXdo  407 KGSIQTYFNVNEQGAVVIRDPQEFSNFQQVAINVSYRASLNDKWNVGLDDPLFTPKSKLS
Vc-ExoY-MARTXdo  412 KGSIQTYFNVNEQGAVVIRDPHEFSNFQQVAINVSYRASLNDKWNVGLDDPLFTPKRKLS
Pa-ExoY-PA14-st  324 SVRF--------DEVCVVADRKSFSLLVECIKGNGYHFTAHPDWNVPLRPSFQ-E----A
Pa-ExoY-PAO1-st  324 SVRF--------DEVCVVADRKSFSLLVECIKGNGYHFTAHPDWNVPLRPSFQ-E----A

Vn-ExoY-MARTXdo  439 HDYLDARDEVAKKLGLTESSKLNGL---------------------
Vv-ExoY-MARTXdo  467 HDFLNAKEEVIKKLSGEVETNVRTTQLLTDNEGL------------
Vc-ExoY-MARTXdo  472 HDFLNAKEEVIKKLSGEVETNVRTTQLLTDNEGEKL----------
Pa-ExoY-PA14-st  371 LDFSNVRSDARRSLAKP--AFPGGMGILPPVQGLPLAGICWAARGS
Pa-ExoY-PAO1-st  371 LDFFQRKV--------------------------------------

S3 Fig. Sequence alignments of MARTX ExoY-Like domains from Vibrio nigripulchritudo (Vn-ExoY-MARTXdo, from UniProt F0V1C5_9VIBR, used in the current study), Vibrio vulnificus (Vn-ExoY-MARTXdo, from UniProt A0A023NA98_VIBVL), Vibrio cholerae (Vc-ExoY-MARTXdo, from UniProt D7H8T5_VIBCL), with ExoY proteins from Pseudomonas aeruginosa strain UCBPP-PA14 (Pa-ExoY-PA14-st, UniProt A0A0H2ZM07_PSEAB), and Pseudomonas aeruginosa strain PAO1 (Pa-ExoY-PAO1-st, UniProt Q9I1S4_PSEAE, used in the current study). Identical and similar residues conserved over 40% of the sequences are shaded in black and gray, respectively. The conserved motifs I (red box) and II (green box) between actin-activated ExoY-like and calmodulin-activated CyaA and EF NC toxins as well as mutations studied in PaExoY and VnExoY are indicated in the sequences. The motif I and II are stabilizing the phosphate, ribose and/or base moieties of the bound nucleotide and its divalent cation in the related class II nucleotidyl cyclase toxins CyaA from Bordetella pertussis and EF from Bacillus anthracis.
