# Supplementary material for: Differential regulation of actin-activated nucleotidyl cyclase virulence factors by filamentous and globular actin
Source: PLoS One. 2018 Nov 12;13(11):e0206133. doi: 10.1371/journal.pone.0206133 (PMC6231621; doi:10.1371/journal.pone.0206133)
Supplement: S1 Table — (DOCX) [file pone.0206133.s004.docx]

**S1 Table. Strains, plasmid, and primers**

|  | **Description** | **Reference** |
| --- | --- | --- |
| **strains** |  |  |
| *S.cerevisiae* MH272-3fα | “Wild-type” strain*, ura3, leu2, his3, trp1, ade2* | (1) |
| *S. cerevisiae* Y70 | *S. cerevisiae* MH272-3fα (YEpGal555) | This study |
| *S. cerevisiae* Y71 | *S. cerevisiae* MH272-3fα (p1648) | This study |
| *S. cerevisiae* Y72 | *S. cerevisiae* MH272-3fα (pB14) | This study |
| *S. cerevisiae* Y73 | *S. cerevisiae* MH272-3fα (pB15) | This study |
| *S. cerevisiae* Y74 | *S. cerevisiae* MH272-3fα (pB16) | This study |
| *S. cerevisiae* Y96 | *S. cerevisiae* MH272-3fα (p1593) | This study |
| *S. cerevisiae* Y97 | *S. cerevisiae* MH272-3fα (p1594) | This study |
| *S. cerevisiae* Y98 | *S. cerevisiae* MH272-3fα (pB46) | This study |
| *S. cerevisiae* Y99 | *S. cerevisiae* MH272-3fα (p1682) | This study |
|  |  |  |
| **plasmids** |  |  |
| YEpGal555 | *E. coli*/*S. cerevisiae* shuttle vector [AmpR/Ade2] | (2) |
| p1648 | Myc-VnExoY in YEpGal555 | This study |
| pB14 | Myc-VnExoY^K117M^ in YEpGal555 | This study |
| pB15 | Myc-VnExoY^K124I^ in YEpGal555 | This study |
| pB16 | Myc-VnExoY^K117M/K124I^ in YEpGal555 | This study |
| p1593 | Myc-PaExoY in YEpGal555 | (3) |
| p1594 | Myc-PaExoY^K81M^ in YEpGal555 | This study |
| pB46 | Myc-PaExoY^K88I^ in YEpGal555 | This study |
| p1682 | Myc-PaExoY^K81M/K88I^ in YEpGal555 | This study |
| pUM460 | inducible expression of ExoY-FH under lambda P*_L_* controlled by temperature sensitive cI (cI857) | (3) |
| pUM498 | ExoY^K81M^-HA in pAG415GAL-ccdB-HA [4] | (3) |
| pUM522 | inducible expression of VnExoY-FH under lambda P*_L_* controlled by temperature sensitive cI (cI857) | (3) |
| pUM530 | VnExoY^K81M^ in pBAD33(4) | This study |
| pUM533 | As pUM460, but VnExoY^K117M^-FH | This study |
| pUM536 | As pUM460, but VnExoY^K117M/K124I^-FH | This study |
| pEA11 | as pGEX-6-P1(5), His-MBP-PaExoY-ST | (3) |
| pLR152 | as pGEX-6-P1, His-MBP-VnExoY-ST | This study |
| pLR153as | As pGEX-Trx (5) Trx-VnExoY | This study |
|  |  |  |
| **primers** |  |  |
| b16 | TATACTCGAGGGCTATAACTATGGTCAGGC |  |
| b17 | TATAGGTACCGAGTCCGTTGAGCTTCGAAG |  |
| b18 | TGTGAAGGCTATTAGCTCTGACTGG |  |
| b19 | CCAGTCAGAGCTAATAGCCTTCACA |  |
| 1259 | CAGCTCGAGCGTATCGACGGTCATCG |  |
| 1260 | ACAAGGTACCTGGGTTGACCTTACGTTG |  |
| b61 | GGTGAAGGGGATAAGCTCGAACTG |  |
| b62 | CAGTTCGAGCTTATCCCCTTCACC |  |
| 1307 | GTGAAGGGGATAAGCTCGAAC |  |
| 1308 | GTTCGAGCTTATCCCCTTCAC |  |
| 1280 | GGAGATATCTCGAGGGCTATAAC |  |
| 40 | TAAAGTATATATGAGTAACCATGGTCTGACAGT |  |
| o59 | GGCGGATCCTATAACTATGGTCAGGCTTTG |  |
| o60 | GGCCTCGAGACCGAGTCCGTTGAGCTTC |  |

**Supplementary References**

1. Peisker K, Braun D, Wolfle T, Hentschel J, Funfschilling U, Fischer G, et al. Ribosome-associated complex binds to ribosomes in close proximity of Rpl31 at the exit of the polypeptide tunnel in yeast. Mol Biol Cell. 2008;19(12):5279-88.

2. Belyy A, Tabakova I, Lang AE, Jank T, Belyi Y, Aktories K. Roles of Asp179 and Glu270 in ADP-Ribosylation of Actin by Clostridium perfringens Iota Toxin. PLoS One. 2015;10(12):e0145708.

3. Belyy A, Raoux-Barbot D, Saveanu C, Namane A, Ogryzko V, Worpenberg L, et al. Actin activates Pseudomonas aeruginosa ExoY nucleotidyl cyclase toxin and ExoY-like effector domains from MARTX toxins. Nat Commun. 2016;7:13582.

4. Guzman LM, Belin D, Carson MJ, Beckwith J. Tight regulation, modulation, and high-level expression by vectors containing the arabinose PBAD promoter. J Bacteriol. 1995;177(14):4121-30.

5. Husson C, Renault L, Didry D, Pantaloni D, Carlier MF. Cordon-Bleu uses WH2 domains as multifunctional dynamizers of actin filament assembly. Mol Cell. 2011;43(3):464-77.
